# Emergence of spontaneous assembly activity in developing neural networks without afferent input

**DOI:** 10.1371/journal.pcbi.1006421

**Published:** 2018-09-28

**Authors:** Marcus A. Triplett, Lilach Avitan, Geoffrey J. Goodhill

**Affiliations:** 1 Queensland Brain Institute, University of Queensland, St Lucia, Queensland, Australia; 2 School of Mathematics and Physics, University of Queensland, St Lucia, Queensland, Australia; Radboud Universiteit Nijmegen, NETHERLANDS

## Abstract

Spontaneous activity is a fundamental characteristic of the developing nervous system. Intriguingly, it often takes the form of multiple structured assemblies of neurons. Such assemblies can form even in the absence of afferent input, for instance in the zebrafish optic tectum after bilateral enucleation early in life. While the development of neural assemblies based on structured afferent input has been theoretically well-studied, it is less clear how they could arise in systems without afferent input. Here we show that a recurrent network of binary threshold neurons with initially random weights can form neural assemblies based on a simple Hebbian learning rule. Over development the network becomes increasingly modular while being driven by initially unstructured spontaneous activity, leading to the emergence of neural assemblies. Surprisingly, the set of neurons making up each assembly then continues to evolve, despite the number of assemblies remaining roughly constant. In the mature network assembly activity builds over several timesteps before the activation of the full assembly, as recently observed in calcium-imaging experiments. Our results show that Hebbian learning is sufficient to explain the emergence of highly structured patterns of neural activity in the absence of structured input.

## Author summary

Even in the absence of sensory stimulation, the developing brain can exhibit highly organised patterns of neural activity. This activity often takes the form of structured assemblies of neurons. Here we draw on calcium imaging experiments in zebrafish larvae to construct a computational model of assembly formation in neural networks without correlated input. Our model shows how a simple learning rule can explain the emergence and dynamics of patterned neural activity in the early nervous system, and predicts a continual reorganisation of assemblies despite maintaining stable statistical properties.

## Introduction

Developing nervous systems exhibit ongoing neural activity even in the absence of sensory stimulation [1]. With recent advances in imaging technology, this spontaneous activity has been shown to be highly organised at the population level [2], and often consists of a number of structured neural assemblies; i.e., groups of neurons that tend to fire together. Neural assemblies have been increasingly interpreted as the basic units of cortical computation and coding, and their presence in spontaneous activity has led to speculation about the contribution of spontaneous activity to neural computation [1]. For instance, spontaneous activity has been hypothesised to interact with evoked activity to affect the representation or processing of information [3, 4]. Spontaneously active neural assemblies can also resemble the population responses evoked by sensory stimuli [5, 6], and thus could contribute to probabilistic inference by acting as a Bayesian prior over possible stimuli in the external environment [5].

The mechanisms surrounding the emergence of structured spontaneous activity have yet to be fully elucidated. Theoretical progress has been made towards understanding how plasticity-driven self-organisation can explain some of the statistical properties of synaptic wiring in cortex [7–13], and on the development and dynamics of structured spontaneous activity in computational models of neural circuits [11, 14–17]. Recently it has been shown how multiple forms of synaptic plasticity and homeostasis can interact to develop neural assemblies from repeated sensory stimulation [18], and how trained memories can be retrieved as the activation of neural assemblies both spontaneously [18] and by partial cues [15] in detailed circuit models. Other models based on spike-timing-dependent plasticity rules have analysed the complementary problem of memory retention. One study of balanced random networks, for example, established that membership in the set of strongest synapses decays exponentially with time [19]. Similarly, receptive field structure in networks with feedforward excitation and lateral inhibition is unstable, with an autocorrelation that decays to zero despite continued stimulation with the same set of stimuli [20].

Surprisingly, however, even when animals are deprived of sensory stimuli during development, spontaneous activity still exhibits a highly structured form [21, 22]. While computational analyses have investigated assembly formation under sensory stimulation, the mechanisms underlying the development of assemblies in systems with no structured afferent input remain poorly understood. Here we focus on constructing a simple computational model that explains how neural assemblies can emerge over developmental timescales in the absence of external input, as recently seen in the developing zebrafish [21, 22]. We describe how a Hebbian plasticity rule that reinforces synchronous neural activity can interact with a simple normalisation rule to reorganise the structure of neural networks into a highly modular state where assemblies activate spontaneously. Rather than focusing on plasticity at a millisecond timescale, we instead consider timesteps on the order of one second. This matches the order of magnitude for both burst-timing-dependent plasticity rules [23] and the temporal resolution of much calcium imaging data for large neural populations, and makes it computationally feasible to track plasticity over developmental timescales. We relate our model to calcium imaging of *in vivo* spontaneous assembly activity, showing how simple mechanisms can explain the emergence and dynamics of structured neural assemblies in the developing brain.

## Results

### The optic tectum develops neural assemblies in the absence of retinal input

**Fig 1:**
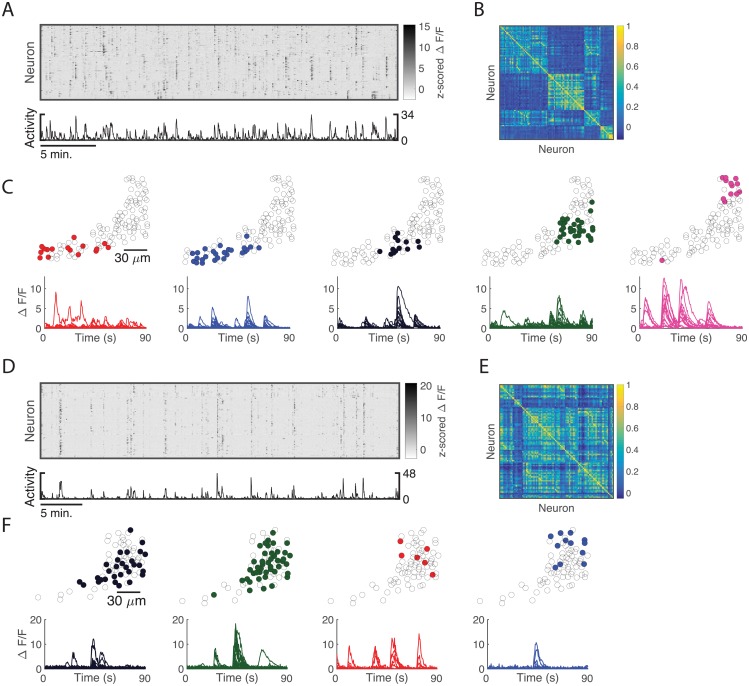
Spontaneous assembly activity in the optic tectum of the larval zebrafish. (A) Fluorescence raster of spontaneous activity in an optic tectum 8 days post-fertilisation (dpf), sorted according to detected assemblies. 114 periventricular neurons were recorded. Shown are the 79 neurons that were members of an assembly. 18 neurons were members of multiple assemblies and thus their rows are repeated in the fluorescence raster. Colour indicates z-scored Δ*F*/*F* for each neuron. Bottom, number of neurons whose Δ*F*/*F* is more than 2 standard deviations above its mean. (B) Correlation matrix of the population of neurons in A. (C) Top, spatial organisation of the five assemblies detected in A. Neurons (not drawn to scale) are coloured by their assigned assembly. Bottom, sample fluorescence traces of spontaneous calcium activity from the assemblies detected in A. Each coloured line corresponds to the activity of a neuron from the associated assembly above. (D)—(F) Same as A-C, but for 64 assembly neurons (76 total, 25 members of multiple assemblies) recorded at 6 dpf from a zebrafish larva bilaterally enucleated at 24 hours post-fertilisation. Even without afferent input neural assemblies form. In this case four assemblies were detected.

Recent studies of population activity in the optic tectum of the larval zebrafish have revealed the presence of recurrent spontaneous assembly activity [21, 22]. Fig 1 shows a comparison of typical assembly activity that emerges in the tectum with and without afferent input, reanalysed from ref. [21]. We applied a spectral clustering-based assembly detection algorithm that extracted neural assemblies from the calcium activity [21], and sorted the fluorescence raster according to the detected assemblies. In normally-reared zebrafish this revealed spontaneous and synchronous bursts of fluorescence from assemblies of neurons, a modular correlation structure, and a small number of spatially structured assemblies (Fig 1A–1C). Remarkably, zebrafish bilaterally enucleated at 24 hours postfertilisation showed qualitatively similar patterns of spontaneous assembly activity in the tectum despite the absence of afferent input (Fig 1E–1H), a result also demonstrated in ref. [22]. Thus, the basic structure of the neural assemblies formed in the zebrafish optic tectum does not depend on afferent input. This raises the question of what mechanisms endogenous to a neural population such as the tectum could explain assembly formation.

### Network modularity determines the structure of spontaneous activity

**Fig 2:**
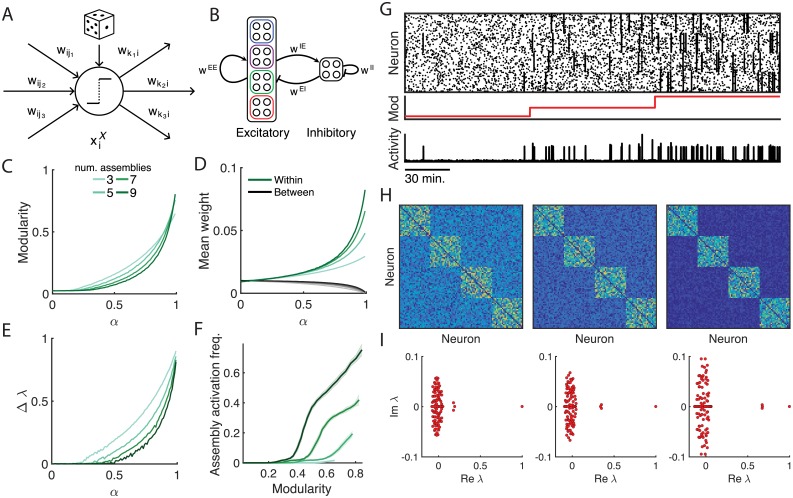
Effects of modularity on the structure of spontaneous activity. (A) Stochastic binary neuron model. Each neuron computes a weighted sum of network activity in the previous timestep. The neuron activates if this sum exceeds the threshold *θ*, or spontaneously by chance. (B) To investigate the effects of a modular weight matrix on spontaneous activity we grouped the excitatory neurons and embedded the group structure into the weight matrix according to the separation parameter *α*. (C) Modularity of the weight matrix as assemblies are embedded more strongly via the separation parameter *α*. (D) The within-assembly connections are strengthened and between-assembly connection-strengths weakened with increasing *α*. (E) Distance between leading eigenvalues and spectral band as assembly embedding is strengthened. (F) Assembly activation frequency as a function of modularity for networks embedded with assemblies via the separation parameter *α*. (G) Spontaneous activity raster of excitatory neurons with embedded assemblies. The network was simulated for a total of 15000 timesteps for *α* = 0.5, 0.7, and 0.9 (red line, middle). As the network modularity increased, so did the rate of assembly activation (bottom trace, showing number of co-active neurons at each timestep). (H) Weight matrices corresponding to the activity raster in C for *α* = 0.5, 0.7, 0.9. (I) Separation of eigenvalues of corresponding weight matrices in H as the embedding of assemblies is strengthened. Vertical and horizontal axes respectively denote the imaginary and real parts of the eigenvalues.

To address this question we simulated spontaneous activity in a recurrent network of binary units (Fig 2A and 2B), where the activities $x_{i}^{\text{E}}$ and $x_{j}^{\text{I}}$ of the excitatory and inhibitory neurons respectively updated according to threshold rules

$$\begin{array}{c} x_{i}^{\text{E}}\left(t+1\right)=\Theta (\sum_{j}w_{ij}^{\text{EE}}x_{j}^{\text{E}}\left(t\right)-\sum_{j}w_{ij}^{\text{EI}}x_{j}^{\text{I}}\left(t\right)+\beta_{i,t}^{\text{E}}-\theta )\\ x_{i}^{\text{I}}\left(t+1\right)=\Theta (\sum_{j}w_{ij}^{\text{IE}}x_{j}^{\text{E}}\left(t\right)-\sum_{j}w_{ij}^{\text{II}}x_{j}^{\text{I}}\left(t\right)+\beta_{i,t}^{\text{I}}-\theta ) \end{array}$$

where Θ is the heaviside step function, *θ* is the activition threshold, and each $\beta_{i,t}^{X}$ is a random variable that drives background spontaneous activity. $\beta_{i,t}^{X}$ takes the value 1 + *θ* (with probability $p_{i}^{X}$) or 0 (with probability $1-p_{i}^{X}$), and to allow for some variation in baseline firing rates we sampled $p_{i}^{X}$ from a Gaussian distribution with mean *μ* and standard deviation *σ* (see Methods for parameter values). In order to draw comparisons between the experimental data and our model, the network initially consisted of 100 excitatory and 25 inhibitory units, which roughly matched the number of neurons recorded experimentally. We also identified each timestep as one second, which matched the order of magnitude of the temporal resolution of the calcium imaging experiments. As the kinetics of genetically-encoded calcium indicators operates on the order of seconds [24], we replaced the rise and decay of the calcium fluorescence by the instantaneous activation of a binary neuron. This also ensured that the network model was tractable enough to simulate for periods of days.

Before studying the effects of Hebbian learning on spontaneous neural activity, we first explored the structure of fixed network wiring required to produce neural assemblies given these activity dynamics. In computational models of neural networks, structured spontaneous activity can result from strongly connected groups of spontaneously active neurons [25]. One approach to characterising the presence of such modular structure in a network is with the graph modularity, a measure that describes how strongly a network can be divided into disjoint modules by comparing the strengths of connections within modules to the expected strengths of connections outside of modules if the network had weights chosen at random [26] (see Methods). We artificially partitioned the set of model neurons into clusters of equal size (Fig 2B) and defined a parameter *α* to control the separation of within-cluster to between-cluster connection strengths. We generated the within-group synaptic weights by sampling at random from *U*(0, 1), the uniform distribution over (0, 1), the between-group synaptic weights from *U*(0, 1 − *α*), and then normalising the weights according to the normalisation procedure described below. As we increased *α* we generated networks of greater modularity and greater within-cluster strengths (Fig 2C and 2D), which strengthened the embedding of the artificial assemblies.

The modularity of a network is closely related to the structure of the eigenvalue spectrum of its weight matrix [27]. Networks with high modularity have more strongly embedded communities, and this is reflected in the spectra of their weight matrices as the separation of eigenvalues into two groups: a continuous “spectral band” comprised of most eigenvalues, and a group of outliers, the number of which is often used to estimate the number of communities present in the network [27]. The leading eigenvalues gradually separated from the spectral band as we strengthened the embedding of the assemblies in the network (Fig 2E). The distance Δλ between the real parts of the eigenvalues in the spectral band and the outliers has previously been related to the presence of assembly activity in balanced networks of integrate-and-fire neurons [28]. Here we show that this relationship also holds for the simplified model dynamics (Fig 2E and 2F). The increasing modularity, within-assembly connection strengths, and separation of eigenvalues caused an amplification of the recurrent excitation within the embedded assemblies and led to their spontaneous activation (Fig 2G–2I). We repeated the analysis for the division of the network into 3 to 9 assemblies and characterized the assembly activation frequency as a function of the modularity (Fig 2F), revealing the existence of a threshold in the graph modularity that a network must exceed before neural assemblies will activate spontaneously.

### Neural assemblies form in the absence of structured afferent input

We next investigated how increases in modularity might arise as a result of self-organisation. We initialised the neural network with random synaptic strengths and modified the excitatory synapses during spontaneous activity according to the covariance learning rule [29]

$$\begin{array}{c} \Delta w_{ij}^{\text{EE}}=\eta \left(x_{i}^{\text{E}}\left(t\right)-\left\langle x_{i}^{\text{E}}\right\rangle \right)\left(x_{j}^{\text{E}}\left(t\right)-\left\langle x_{j}^{\text{E}}\right\rangle \right), \end{array}$$

which updated the strength of the connection $w_{ij}^{\text{EE}}$ between excitatory units *i* and *j* at rate *η* (where 〈⋅〉 denotes averaging over time). We enforced a lower saturation constraint to prevent the excitatory connections from becoming negative, and then defined a normalisation rule to regulate the growth of the synaptic weights. A common consequence of Hebbian plasticity is the emergence of rich-get-richer dynamics [10], where a small fraction of neurons acquire increasingly stronger outgoing connections and come to dominate the population activity. To prevent this we presynaptically normalised the connections leaving each neuron *j* by setting ${\tilde{w}}_{ij}^{\text{EE}}=w_{ij}^{\text{EE}}/\sum_{k}w_{kj}^{\text{EE}}$. In our model *θ* was the proportion of the total possible synaptic input that a neuron needed to activate, so we next postsynaptically normalised the modified connections with $w_{ij}^{\text{EE}}={\tilde{w}}_{ij}^{\text{EE}}/\sum_{k}{\tilde{w}}_{ik}^{\text{EE}}$ to ensure that this proportion was constant across all iterations. All connections other than EE-type connections were kept fixed throughout the simulation (see Discussion).

**Fig 3:**
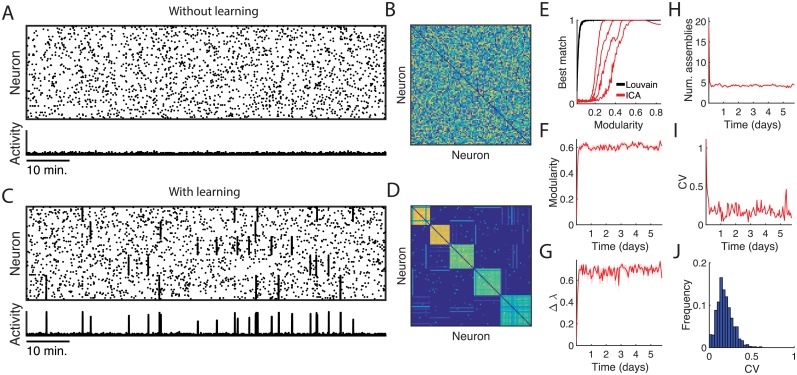
Effects of Hebbian learning on the structure of spontaneous activity. (A) Spontaneous activity in the neural network without Hebbian learning. (B) Matrix of uniformly sampled and normalized synaptic weights. (C) Spontaneous activity in the neural network with Hebbian learning, recorded once the network converged to a stable number of assemblies and then sorted according to detected assemblies. Neurons self-organised into strongly connected clusters and activated spontaneously as neural assemblies. (D) Weight matrix of mature neural network with Hebbian learning. (E) Assembly reconstruction task. Assemblies are embedded into weight matrices according to the separation parameter *α*. The Louvain method mines the weight matrix directly for community structure to recover the embedded assemblies. The weight matrices were used to simulate 10,000 seconds of spontaneous activity which the ICA method mines for assemblies. Graph shows the best match performance of the Louvain method (black) and the ICA method (red) in recovering assemblies embedded with increasing modularity. Each line corresponds to the performance of a method in recovering assemblies when different numbers of assemblies have been embedded, where the left-most line is for simulations with 9 assemblies, and the right-most line with three assemblies. (F) Network modularity over development. (G) Distance between leading eigenvalues and the spectral band over development. (H) Number of assemblies detected from the synaptic weight matrix over development. (I) Coefficient of variation of assembly sizes for a single simulation. (J) Distribution of coefficients of variation of assembly sizes for networks that were simulated for 100,000 timesteps (approximately 28 hours model time). Each simulation was initialised with a random synaptic weight matrix. Shaded error bars in panels F-H indicate SEM over 10 trials.

The interaction between the covariance learning rule and the pre- and post-synaptic normalisation rule caused the spontaneously activating neurons to organise into assemblies that activated in synchronous bursts (Fig 3A and 3C). The synaptic weight matrix of the neural network reveals how the plasticity rule reshaped the structural connectivity of the network into distinct modules (Fig 3B and 3D). We then extracted modules from the weight matrix using a modularity-based community detection algorithm (the Louvain method [30]).

To confirm that the modules detected from the synaptic weight matrix coincided with the assemblies visible in the event raster, we compared the ability of a standard assembly detection algorithm based on independent components analysis [31] with the Louvain method in a task to recover artificially defined assemblies of varying size and with a range of imposed modularities. We generated weight matrices that embedded assemblies with increasing modularity using the separation parameter *α*. For each generated weight matrix we simulated 10,000 seconds of spontaneous activity and applied ICA to recover the artificially embedded assemblies from the activity raster, and the Louvain method directly to the weight matrix. To measure how well the recovered assemblies corresponded to the embedded assemblies we used a variant of the best match score [32], which has been used previously to estimate the accuracy of assembly detection algorithms [21]. We measured the performance of an assembly detection method by comparing the detected assemblies to the artificial assemblies with the best match score, where the assembly detection algorithm perfectly recovered the embedded assemblies if they shared a best match score of 1. In every condition, the Louvain method operating on the weight matrix performed at least as well as ICA in recovering the predefined assemblies (Fig 3E), confirming that extracting modules directly from the weight matrix via the Louvain method is an effective method for detecting assembly structure.

We tracked the development of the network’s structural properties as we simulated spontaneous activity (Fig 3). The evolution of the network tended to display two phases, consisting of an initial assembly formation phase where the network modularity increased monotonically and the leading eigenvalues separated from the spectral band (Fig 3F and 3G) indicating that assemblies were being embedded into the network, followed by a stable phase where the number of assemblies remained approximately constant (Fig 3H) and assembly activity corresponded to the attractor states of a multistable dynamical system [33].

We used the coefficient of variation (CV) to estimate the dispersion of the set of assembly sizes that the network generated. A CV near 1 indicates that the assembly sizes are highly heterogeneous, whereas a CV near 0 indicates a homogeneous distribution of assembly sizes. For a single simulation the assembly size CV followed the basic trajectory of the number of assemblies, decreasing monotonically before reaching a stable range of values (Fig 3I). We recorded the CV for 1000 independent simulations of 100,000 time steps of network development (approximately 28 hours model time), starting from randomly generated synaptic weight matrices each trial. This revealed the variability in the dispersion measures for the assembly sizes (Fig 3J). At 100,000 time steps the model had a mean assembly size CV of 0.19, suggesting that, while there is some variability, most assemblies tended to be of a similar size.

### Structural analysis of network development

**Fig 4:**
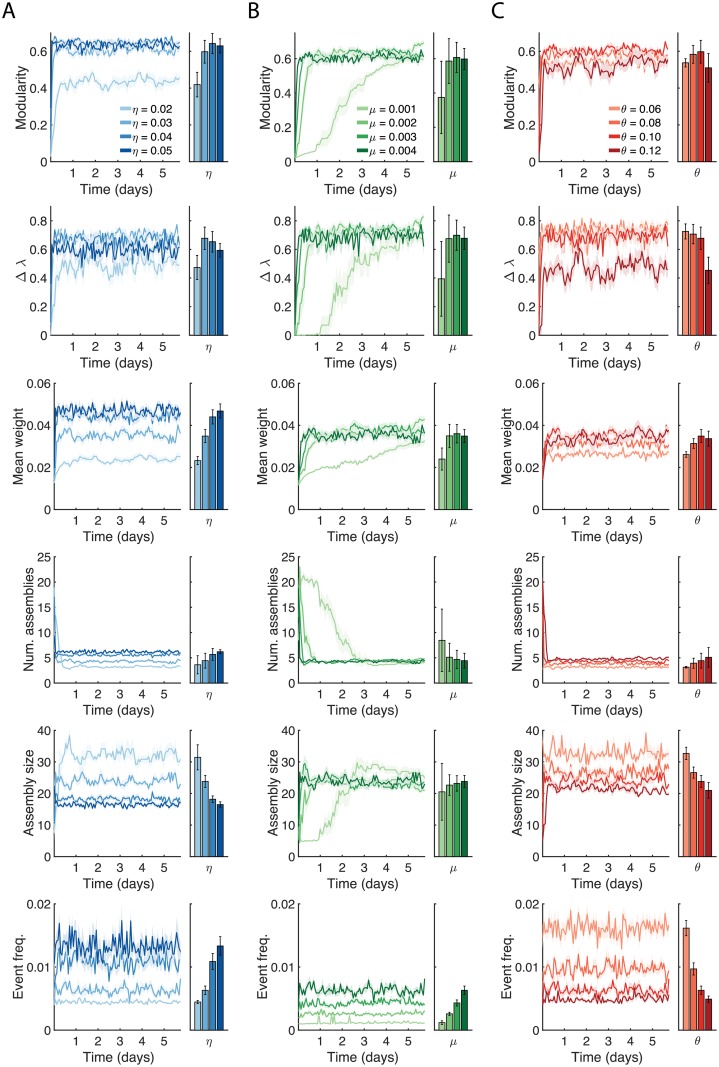
Analysis of network structure over development. (A) Effects of varying the learning rate *η* on network modularity, Δλ, mean within-assembly weight, number of detected assemblies, mean size of detected assemblies, and mean event frequencies. Increasing learning rates generate networks with greater modularity and more assemblies that are more strongly connected. Shaded error bars indicate the SEM over 10 trials. Bar graphs indicate means over the course of the simulation, with error bars indicating 1 standard deviation of the mean trace. (B) Same as A, but for the mean baseline rate of activation *μ*. Increased *μ* accelerates the formation of assemblies, with little effect on the stable structural properties. (C) Same as A, but for the activation threshold *θ*. Similar to increases in the learning rate, increases in the activation threshold principally affect the stable structural properties.

We then performed a more complete analysis of the structural properties of the network during its development. The learning rate *η* controlled the rate of structural modification in the system, so one could expect that greater learning rates should allow the network to approach a stable modularity more rapidly. We found that greater *η* induced greater numbers of assemblies with higher modularity and stronger within-assembly weights (Fig 4A), but with little effect on the rate of network development; i.e., how quickly the network converged to a stable number of assemblies.

The spontaneous activation rates $p_{i}^{X}$ are drawn from a normal distribution with a mean rate *μ*. Surprisingly, when we varied *μ* we found that structural properties such as the modularity, number of assemblies, and within-cluster weights were unaffected in their stable values, but were strongly affected in their time required to stabilize (Fig 4B). The delayed learning time for small *μ* is likely a result of the fewer activations providing fewer opportunities for the Hebbian learning rule to modify the network structure, causing a prolonged assembly formation phase, but without affecting the stable structural properties.

In our model a neuron’s excitability is inversely related to the activation threshold *θ*. We studied the development of spontaneous activity when *θ* deviated from its default value of 0.1. More excitable networks had greater mean event frequencies (Fig 4C) but developed fewer assemblies that tended to be more weakly connected. Thus *η* and *θ* regulated the structural properties of the mature architecture (number of assemblies, assembly size, modularity, within-assembly synaptic strengths) with only minor effects on the temporal evolution of the network, while *μ* governed the timescale of the network evolution, leaving the stable structural properties relatively unaffected.

### Emergent assemblies are continuously reorganised over development

**Fig 5:**
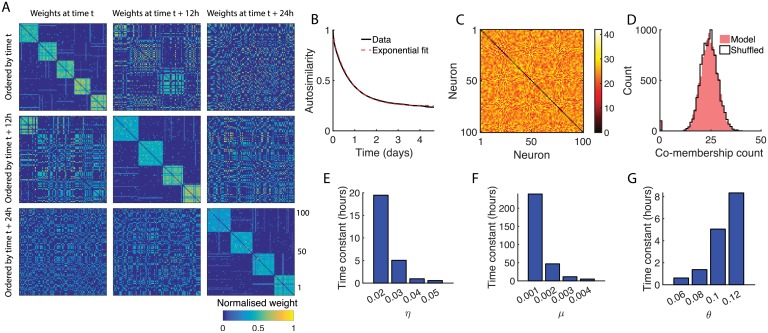
Reorganisation of neural assemblies. (A) Synaptic weight matrices sampled at 12 hour intervals show a gradual reorganisation of the network structure. (B) Autosimilarity decay curve for *η* = 0.02 and fitted exponential. (C) Matrix recording the number of times each pair of neurons appeared in the same assembly over 200 days. Diagonal elements set to zero for ease of visualisation. The Louvain method for community detection fails to identify any strong modular structure. (D) Histogram of co-membership counts in C. Shuffled data presented for comparison, obtained by randomly reassigning neurons to assemblies in each sample. (E) Time constants for exponential fits of autosimilarity decay curves with increasing *η*. (F) Same as E but for *μ*. (G) Same as E but for *θ*. Coefficient of determination *R*^2^ = 1 for all fitted exponentials.

Spontaneous activity typically degrades structured patterns of connectivity in neural networks with plastic synapses. However, it was recently shown that spontaneous activity can be critical to reinforcing a learned network architecture [18, 34]. Observing that the number of assemblies in our network model became approximately constant (Fig 4), we asked whether the spontaneously developed neural assemblies were stable over time, or if they degraded and were continuously replaced by new assembly structures. Remarkably, we found that despite the roughly constant number of assemblies, the composition of each assembly was constantly changing (Fig 5A). We recorded the set of assemblies present at regular intervals and defined an index to measure the similarity of the assemblies present at two different time points, which we refer to as the autosimilarity. The autosimilarity was defined as a function of the time Δ*t* between sampling timepoints via the best match score, and took values between 0 (if the mean best match score between assemblies sampled with an interval Δ*t* is 0) and 1 (if the mean best match score is 1, see Methods).

The autosimilarity decayed exponentially as assemblies were sampled at greater intervals (Fig 5B). We considered whether assemblies eventually became randomly reorganised, or if there were ‘core’ groups of neurons that were maintained despite the reorganisation. Following the initial assembly formation period, we simulated the network for 200 days (model time) and sampled the assemblies every 2 days. This sampling interval was sufficiently long to allow the autosimilarity to decay to its baseline asymptote. We then recorded how many times every pair of neurons was assigned to the same assembly in a co-membership matrix (Fig 5C and 5D). The Louvain method for community detection failed to identify any strong modular structure in the matrix, suggesting that the network does not retain subsets of neurons during its reorganisation.

For each autosimilarity decay curve we fit an exponential of the form

$$\begin{array}{c} y=\beta_{\text{baseline}}+\beta_{\text{amplitude}}\text{exp}\left(-\Delta t/\beta_{\text{decay}}\right) \end{array}$$

and tracked the time constants *β*_decay_ as we varied the model parameters. We found that the time taken for a set of assemblies to decay was highly sensitive to the learning rate, baseline rate of activation, and excitability (Fig 5E–5G), sometimes changing by an order of magnitude with a small deviation in a single parameter. While high learning rates generated more assemblies with greater within-assembly connection strengths, the increased rate of structural modification due to large *η* led to more rapid assembly degradation (Fig 5E).

When we examined the autosimilarity decay for *μ* = 0.001 we saw a substantial increase in the decay time constant (Fig 5F), due to structural modification being greatly prolonged at low rates of baseline activity (Fig 4B). As we increased the baseline rate of activation the emergent assemblies maintained similar within-assembly connection strengths, but degraded much more rapidly (Fig 5F) as a result of the synaptic structure being modified at a much higher frequency caused by the increase in the frequency of synchronous events. Next, we studied how network excitability affected autosimilarity via the threshold parameter *θ*. At low *θ* the network was highly excitable, which increased the event frequency due to the influence of lateral connections, causing rapid and continual modification to the assembly structure (Fig 5G). High levels of *θ* reduced the influence of the recurrent input, and thereby reduced the frequency of synchronous events, which greatly extended the autosimilarity decay time (Fig 5G).

### Assembly activation in the mature network

**Fig 6:**
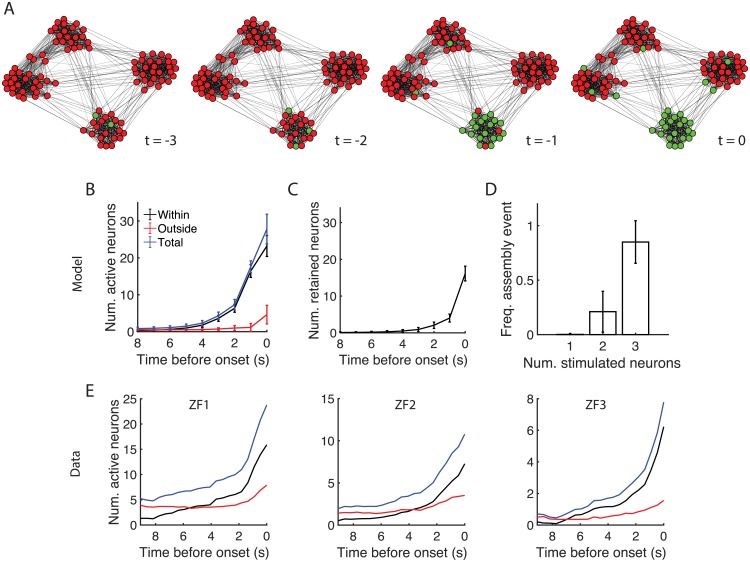
Behaviour of mature neural network with frozen synaptic weights. (A) Example assembly activation event visualised via the Fruchterman-Reingold algorithm [61]. Event onset occurs at time *t* = 0. Green (respectively, red) vertices correspond to active (inactive) model neurons. (B) Average number of neurons active within the assembly (black), outside the assembly (red), and in total (blue) preceding the activation of the complete assembly. Error bars denote one standard deviation over 100 simulations. (C) Mean number of neurons within an assembly that were active at time *t* − 1 and remained active at time *t*. (D) Proportion of stimulated groups of neurons within an assembly that resulted in an assembly activation. (E) Average number of active neurons preceding an assembly event for three example zebrafish larvae (cf. panel B). ZF1 corresponds to the example zebrafish in Fig 1D–1F.

What are the detailed temporal dynamics of assembly activity in the mature network? To investigate this we simulated the development of spontaneous activity in the network for 100,000 timesteps, and then froze the synaptic weights and probed the assembly activation process in the fixed network. Fig 6A shows a typical assembly activation event. Here the coincidental activation of two neurons within an assembly initiated a sequence of activity that resulted in the activation of the complete assembly. We characterised this assembly activation process by calculating the average number of neurons active preceding the event onset (Fig 6B). This revealed a steady build-up of activity within the assembly prior to the event onset, without recruiting many neurons from other assemblies. We then considered whether the neurons that were active leading up to the assembly events were persistently active, in which case the set of active neurons would simply accumulate until the event onset, or whether distinct groups of neurons were active at each timestep. We calculated the number of neurons that were active at both timesteps *t* − 1 and *t* as we varied *t* over the time window preceding each assembly event. This showed that neurons tended to not stay persistently active during the build up to the assembly event, but rather mostly disjoint groups of neurons activated in sequence up until the last two timesteps (Fig 6C, c.f. Fig 6A).

In order to establish how many neurons were required to trigger a complete assembly event we next disabled spontaneous background activity in the network and manually activated sets of neurons. We targeted in turn all possible combinations of 1, 2 or 3 neurons within an assembly and recorded the ratio of these combinations that resulted in the complete activation of the assembly within an interval of 20 timesteps (Fig 6D). While only 0.2% of individual neurons could trigger an assembly event, this rose to 21% and 85% for neuron pairs and triples respectively, indicating that most combinations of 3 neurons were sufficient.

We then compared these model results to the experimental data. In particular, we computed the average number of active neurons preceding an assembly event in the zebrafish recordings in analogy to Fig 6B (see Methods for the definition of event onset times). The experimental data was qualitatively similar to the simulated data (Fig 6E). In the tectum, assembly events had a prolonged build-up and recruited increasing numbers of within-assembly neurons prior to the event onset, with little recruitment of neurons outside of the assembly (Fig 6E). This occurred on a timescale comparable to the activation process in the simulated data, which provides support for the choice of timescale in the computational model. An analogue to Fig 6C is difficult to obtain from the experimental data since the kinetics of the calcium indicator caused active neurons to remain active for several seconds, inflating the estimates of how many neurons were active at successive timesteps.

### Locally connected neurons form spatially organised assemblies

**Fig 7:**
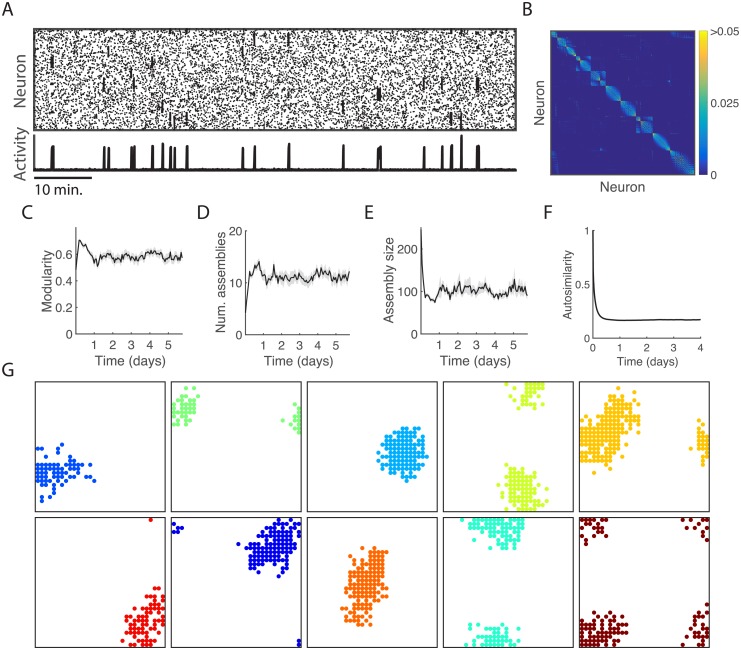
Assemblies generated by the neural network with distance-dependent connectivity and periodic boundary conditions. (A) Spontaneous activity in the Hebbian network model with 1024 excitatory neurons and distance-dependent connection strengths. (B) Synaptic weight matrix corresponding to the neurons in A. (C)—(F) Modularity, number of assemblies, assembly size, and autosimilarity show normal behaviour in the larger network with spatial constraints. (G) Spatial organisation of spontaneously active neural assemblies. Neurons are coloured according to their assembly membership.

The locally structured spatial organisation of assemblies in the optic tectum could potentially result from nearby neurons receiving correlated input due to the topography of the retinotectal projection [35]. However, the persistence of spatiotemporal assembly structure in systems without afferent input suggests that this emergent spatial pattern could also be the result of an endogenous tectal mechanism. In the mammalian nervous system, lateral connections are hypothesised to substantially influence the development and structure of cortical maps, and many theoretical studies of cortical map formation model the source of excitatory synaptic input by neurons at anatomically short distances [36, 37]. We therefore investigated local excitatory connectivity as a mechanism to explain the emergence of the spatial structure of neural assemblies. We assigned each excitatory neuron coordinates in a two-dimensional grid and constrained the strength of their connections by a Gaussian function of the distance between them (see Methods). In order to improve the visualisation of the network’s spatial properties we increased the size of the network to 1024 excitatory neurons and 256 inhibitory neurons. Inhibitory neurons were not assigned spatial locations and innervated every excitatory neuron, similar to the dense and non-specific patterns of inhibitory connections observed in cortex [38]. Additionally, to eliminate edge effects we imposed periodic boundary conditions. We found that enforcing these local excitatory interactions caused neurons to self-organise into spatially structured assemblies that were spontaneously active (Fig 7A and 7B). We verified that the larger model with spatial constraints exhibited a developmental profile in line with our earlier results, such as convergence to a stable range of values for various network metrics (Fig 7C–7E), and had an autosimilarity decay time constant within the range previously identified (Fig 7F, c.f. Fig 5E–5G). For some assemblies the activation sequence was more prolonged than in the smaller network (Fig 7A). The emergent assemblies tiled the two-dimensional surface (Fig 7G), resembling the organisation of assemblies in the optic tectum (Fig 1). Our model can thus explain how a simple, biologically plausible mechanism leads to the emergence of realistic assembly structure.

## Discussion

Inspired by recent data from the zebrafish optic tectum, we have proposed a theoretical account of the emergence of assembly structure in developing neural systems without afferent sensory input. We characterised the presence of assembly structure in the synaptic weight matrix using the graph modularity, a measure often used to identify communities in large social networks. Consistent with theory on the eigenvalue spectra of random graphs, we found that the development of assembly structure was reflected in the gradual separation of eigenvalues in the spectrum of the synaptic weight matrix. By coupling a standard Hebbian learning rule with a simple weight normalisation rule, we demonstrated how a recurrent neural network could transform independent spontaneously activating neurons into strongly connected assemblies that activated in synchronous bursts. The network exhibited an initial assembly formation phase where the modularity increased monotonically and the leading eigenvalues separated from the spectral band, followed by a more stable phase where the number of assemblies was approximately constant. The simplicity of our model allowed us to isolate fundamental parameters that govern assembly formation and modification over long timescales, and we showed how these parameters affect the network structure and timescale of development. We characterised the assembly activation process in the mature network and showed how this qualitatively resembled assembly activation in the experimental data.

Previous models of assembly formation have focused on identifying crucial physiological processes at the millisecond timescale needed for stable assembly formation from correlated input. The model of ref. [18] produced spontaneous assembly activity, and found realistic STDP, homeostatic inhibitory plasticity, and balanced excitation and inhibition to be critical elements of the model, which addressed the transient spontaneous reactivation of trained assemblies in cortical networks that reflected previously experienced stimuli. A similar model [15] showed that multiple forms of plasticity operating in concert could embed stable assemblies that could be retrieved over long timescales. However, in this model one of the trained assemblies is always active, and the network switches discretely between these high activity states. While the spontaneous formation of coactive groups of neurons from spike-timing plasticity rules was reported in a previous model [39], our study differs from this model in several important ways. In ref. [39], a neuron was a member of a neuronal group (analogous to an assembly) if it was driven to spike by the synchronous arrival of spikes from presynaptic neurons already belonging to the group, starting from an initial fixed neuron. In this way the activation of neuronal groups was sequential. Neuronal group identification was then performed combinatorially by cycling through candidate neurons and checking the membership criterion. By contrast, in our model assembly activation was synchronous, and our characterisation of assembly structure with the synaptic modularity naturally lended itself to modularity-based algorithms for assembly extraction. Importantly, the emergent structure of spontaneous activity in our model resembled experimentally observed patterns of neural activity in zebrafish larvae.

Synaptic normalisation is a basic computation observed across a variety of brain areas and nervous systems [40]. Experimental work has shown the conservation of total synaptic weight accompanies activity-dependent potentiation or depression in, for example, synaptic inputs to intercalated neurons of the amygdala [41] and in hippocampal slice cultures [42]. Our model relies on the pre- and postsynaptic redistribution of connection strengths to conserve the total quantity of synaptic weight in the network, constrain the growth of individual connection strengths, and encourage competition among synapses. Earlier models of assembly formation either performed excitatory postsynaptic normalisation [18] or enforced hard constraints on synaptic weights to control their growth [15]. Another previous study examined how feedforward chains of activity (in contrast to synchronous assembly activity) can develop from initially random connections by combining STDP with a pre- and postsynaptic normalisation rule in a network of binary neurons similar to ours [43].

The timescale of the covariance plasticity rule could be most closely related to the time constants involved in NMDA receptor activity, and our plasticity rule could loosely correspond to recently discovered rules for burst-dependent plasticity in the lateral geniculate nucleus [44] and cerebellum [45] which appear to operate on the order of seconds [23]. Our model does not incorporate recently studied rules for homeostatic inhibitory plasticity [46], which, in earlier models of assembly formation [15, 18], were required to maintain network stability while assemblies were being embedded. The temporal resolution of our model allowed the network to maintain stability without homeostatic mechanisms like inhibitory plasticity that act at a timescale on the order of milliseconds; however, such mechanisms may be important for models based on more physiologically detailed neurons.

Our model shows that assemblies can form from endogenously generated spontaneous activity in the absence of any afferent input. *In vitro* cultures of dissociated cells certainly operate in this regime. Networks of dissociated hippocampal neurons spontaneously release glutamate at excitatory synapses, and a number of recent studies have recorded the emergence of coordinated spontaneous activity in the development of hippocampal cultures [47–50]. It is, however, currently unclear the extent that afferent input is fully absent in any particular *in vivo* system. In the larval zebrafish example (Fig 1), while early bilateral enucleation removed the main afferent input to the tectum, it has recently been shown that there are also tectal inputs from the lateral line and auditory systems [51]. Afferent input could be definitively removed by explanting the tectum and recording the development of tectal spontaneous activity *ex vivo*. This would be challenging, however, as tectal removal would have to occur before the first retinal axons enter the tectum at approximately 2 dpf, and possibly earlier depending on other sources of afferent input. Explanting mammalian cortex prior to its innervation by any afferent input would likely be even more challenging.

While assemblies can develop without input, perhaps more normally they develop through a combination of mechanisms, with afferent input sometimes playing a more modulatory rather than deterministic role. This would provide a powerful way of allowing mechanisms learned over evolutionary timescales (i.e., formation of assemblies using only endogenous activity) to interact with the specific features of the environment in which the animal develops. Alternatively, the endogenous development of assemblies could be a simple by-product of mechanisms of activity-dependent circuit development primarily established for processing afferent input.

Previous studies of the development of neural activity in the cortex have shown that, while spontaneous activity can reflect the statistics of natural stimuli presented to an animal [5], spontaneous cortical activity can also exhibit structured correlations prior to eye opening. Spontaneous waves of activity originate in the retina and propagate through the thalamus and cortex [52], and are thought to play a critical role in the activity-dependent refinement of cortical connections in early development. Wide field calcium imaging has revealed that the spatial structure of correlated spontaneous activity persists after blocking feedforward synaptic input from the retina and the lateral geniculate nucleus [53], and an enucleation study of developing mice showed that highly synchronised calcium events in L2/3 occur independently of retinal inputs [54], indicating that structured spontaneous activity could be endogenously generated by cortical circuits. Another recent study has suggested that the spatial structure of spontaneous activity in primary visual cortex prior to eye-opening follows a mexican-hat profile [55]. This contrasts with the simple decay with distance we used to reproduce the spatially compact assemblies seen in the optic tectum. However, our model could be adapted to the case of activity in the developing visual cortex by including mexican-hat-type connectivity schemes, similar to those often used in computational models of cortical map formation [36, 56].

A particularly surprising property of our model was that, despite the number of emergent assemblies remaining approximately constant following the formation phase, the assemblies were then continuously modified. Since synchronous events are rare, for the plasticity rule to generate modular subnetworks it must make relatively large modifications to the network structure whenever there is coincidental neural activity. The modular structure will then continue to be modified even once the network has passed into the stable phase. Thus a network that is plastic enough to generate assemblies without correlated afferent input may not be capable of stabilising them unless structural modification is suppressed, for instance by the closure of a critical period in early development [57].

The continual reorganisation of neural assemblies over development is a novel prediction of the model that has yet to be tested experimentally in zebrafish. This would require chronic imaging of the same set of identified tectal neurons over many hours, or repeatedly on subsequent days. In recent years this kind of chronic recording with calcium imaging has become possible in adult mice. Experiments in the parietal cortex show that patterns of population activity representing task features and associated learned behaviours are stable within single days, but are continuously reorganised over weeks without an appreciable deterioration in the animal’s performance [58]. Similarly, the set of neurons forming hippocampal place codes changes continuously while the ongoing representation of space is accurately preserved at the population level [59]. Such observations suggest a strategy for maintaining learned representations while flexibly incorporating novel associations. In the developing optic tectum, the continual reorganisation of spontaneously generated assemblies in the absence of structured afferent input could act as a mechanism for exploring potential network configurations. This would offer the system a computational advantage in optimally adapting to its particular environment.

## Materials and methods

### Ethics statement

All procedures were performed with approval from The University of Queensland Animal Ethics Committee (approval certificate number QBI/152/16/ARC).

### Zebrafish

Nacre zebrafish (*Danio rerio*) embryos expressing *elavl3:H2B-GCaMP6s*, of either sex, were collected and raised according to established procedures [60] and kept under a 14/10 hr on/off light cycle except where otherwise indicated. Larvae were fed live rotifers (*Brachionus plicatilis*) from 5 days postfertilisation unless otherwise indicated.

### Calcium imaging

The procedures for the calcium imaging experiments are reported in ref. [21].

### Neural network model

#### Network

The model consisted of a completely connected recurrent network of excitatory and inhibitory binary neurons, whose respective activity states updated according to the threshold rules

$$\begin{array}{c} x_{i}^{\text{E}}\left(t+1\right)=\Theta (\sum_{j}w_{ij}^{\text{EE}}x_{j}^{\text{E}}\left(t\right)-\sum_{j}w_{ij}^{\text{EI}}x_{j}^{\text{I}}\left(t\right)+\beta_{i,t}^{\text{E}}-\theta )\\ x_{i}^{\text{I}}\left(t+1\right)=\Theta (\sum_{j}w_{ij}^{\text{IE}}x_{j}^{\text{E}}\left(t\right)-\sum_{j}w_{ij}^{\text{II}}x_{j}^{\text{I}}\left(t\right)+\beta_{i,t}^{\text{I}}-\theta ) \end{array}$$

where Θ is the heaviside step function and *θ* is the activition threshold. The noise variables $\beta_{i,t}^{X}$ were defined by

$$\begin{array}{c} \beta_{i,t}^{X}=\{\begin{array}{cc} 1+\theta & \text{with}\;\text{probability}\;p_{i}^{X}\\ 0 & \text{with}\;\text{probability}\;1-p_{i}^{X} \end{array} \end{array}$$

where $p_{i}^{X}∼N\left(\mu ,\sigma^{2}\right)$ and *μ* and *σ* are model parameters. Autapses (i.e., self-connections) were not allowed. In all of our simulations we used a 4:1 ratio of excitatory to inhibitory neurons.

#### Covariance plasticity rule

The excitatory synapses were modified at every timestep *t* according to the covariance learning rule

$$\begin{array}{c} \Delta w_{ij}^{\text{EE}}\left(t\right)=\eta \left(x_{i}^{\text{E}}\left(t\right)-{\left\langle x_{i}^{\text{E}}\right\rangle }_{t}\right)\left(x_{j}^{\text{E}}\left(t\right)-{\left\langle x_{j}^{\text{E}}\right\rangle }_{t}\right), \end{array}$$

where ${\langle x_{i}^{X}\rangle }_{t}=\frac{1}{t}\sum_{\tau =1}^{t}x_{i}^{X}\left(\tau \right)$ denotes averaging over all preceding timesteps. Each neuron can implement the averaging ‘online’ by storing the previous mean activity state ${\langle x_{i}^{X}\rangle }_{t-1}$ and updating this as ${\langle x_{i}^{X}\rangle }_{t}=\frac{1}{t}(\left(t-1\right){\langle x_{i}^{X}\rangle }_{t-1}+x_{i}^{X}\left(t\right))$.

#### Normalisation rule

Every model neuron approximately maintained a constant sum of both their efferent and afferent weights with a two-step normalisation procedure:

$$\begin{array}{ccc} {\tilde{w}}_{ij}^{\text{EE}}\left(t+1\right)=w_{ij}^{\text{EE}}\left(t\right)/\sum_{k}w_{kj}^{\text{EE}}\left(t\right) & \;\text{for}\;\text{all}\;i,j & \text{(presynaptic)}\\ w_{ij}^{\text{EE}}\left(t+1\right)={\tilde{w}}_{ij}^{\text{EE}}\left(t+1\right)/\sum_{k}{\tilde{w}}_{ik}^{\text{EE}}\left(t+1\right) & \;\text{for}\;\text{all}\;i,j & \text{(postsynaptic)} \end{array}$$

The normalisation rule was applied at every timestep following the application of the covariance plasticity rule.

#### Initialisation

For each simulation, all connections in the network were initialised by randomly sampling their strengths uniformly between 0 and 1. All connections were then normalised according to the normalisation rule. All connections other than EE-type connections were fixed throughout the simulation.

#### Connection strength bounds

The excitatory connections were subject to a lower saturation constraint to prevent them from becoming negative, and the above normalisation procedure implicitly defined an upper constraint on the synaptic weight of 1.

#### Distance-dependent connectivity

We introduced a spatial dimension into the model by arranging units on a square grid of size *N*_*e*_. Each neuron *i* ∈ {1, …, *N*_*e*_} was assigned a corresponding coordinate (*x*_*i*_, *y*_*i*_), and the distance between neurons *i* and *j* was given by the Euclidean distance of their coordinates. We spatially constrained the connections by imposing a maximum connection strength *w*^max^(*d*) defined as

$$\begin{array}{c} w^{\text{max}}\left(d\right)=a\text{exp}(-\frac{d^{2}}{2\kappa^{2}}) \end{array}$$

where *d* is the distance between units assuming periodic boundary conditions.

Parameter values for the basic neural network model and the model with distance-dependent connection strengths are given in Tables 1 and 2. Matlab code for simulating the model is available at https://github.com/marcustriplett/assembly_formation_model.

**Table 1:** Default parameter values for the Hebbian network model.

Basic network model		
*N*_*n*_	Population size	125
*N*_*e*_	Number of excitatory units	100
*N*_*i*_	Number of inhibitory units	25
*η*	Learning rate	3 × 10^−2^
*μ*	Mean baseline activation rate	4 × 10^−3^
*σ*	Standard deviation of baseline activation rate	3 × 10^−4^
*θ*	Activation threshold	0.1

**Table 2:** Parameter values for the network model with distance-dependent connectivity and periodic boundary conditions.

Locally connected model		
*N*_*n*_	Population size	1280
*N*_*e*_	Number of excitatory units	1024
*N*_*i*_	Number of inhibitory units	256
*η*	Learning rate	4 × 10^−2^
*μ*	Mean baseline activation rate	2 × 10^−3^
*σ*	Standard deviation of baseline activation rate	3 × 10^−4^
*θ*	Activation threshold	0.1
*a*	Amplitude of Gaussian connectivity function	0.1
*κ*	Width of Gaussian connectivity function	3

### Network modularity

The modularity is a graph-theoretic measure that describes how strongly a network can be divided into disjoint modules by comparing the strengths of connections within modules to the expected strengths of connections outside of modules if the network had weights chosen at random [26]. Formally, the modularity *Q* of the synaptic weight matrix *w*^*EE*^ is a measure of the strength of its division into clusters {*c*_*i*_}_*i*_. The Louvain method symmetrises the weight matrix by putting

$$\begin{array}{c} \tilde{w}=w^{EE}+{\left(w^{EE}\right)}^{T}. \end{array}$$

This modification of the weight matrix is reasonable for our network because the plasticity rule acts symmetrically and the normalisation rule distributes synaptic weight both pre- and post-synaptically, causing the weight matrices generated by the model to be highly symmetric (cf. Figs 3, 5 and 7). Let $m=\sum_{i,j}{\tilde{w}}_{ij}$ be the sum of the synaptic strengths in the symmetrised network and $k_{i}=\sum_{j}{\tilde{w}}_{j}$ be the sum of incoming and outgoing connections for neuron *i*. For a general weighted network, the modularity given a particular division of the network into clusters is calculated (up to a multiplicative constant) by summing the difference between the strength of the connection between *i* and *j* and the expected strength of the connection if the weights were chosen at random, ${\tilde{w}}_{ij}-\frac{k_{i}k_{j}}{2m}$, over all pairs *i*, *j* within the same cluster *c*_*k*_. The complete modularity of $\tilde{w}$ is then calculated by maximising this value over all the divisions of the network into clusters

$$\begin{array}{c} Q^{\tilde{w}}=\underset{c}{\text{max}}\frac{1}{2m}\sum_{i,j}({\tilde{w}}_{ij}-\frac{k_{i}k_{j}}{2m})\delta \left(c_{i},c_{j}\right), \end{array}$$

where *δ*(*x*_1_, *x*_2_) = 1 if *x*_1_ = *x*_2_ and 0 otherwise, and each *c*_*i*_ is a community in ***c***.

### Assembly detection

The Louvain method extracts communities from the synaptic weight matrix *w* by selecting the network division ***c*** that maximises $Q^{\tilde{w}}$. While the calculation of the exact network modularity is NP-hard, the Louvain method uses an iterative greedy optimization algorithm to approximate the modularity (code obtained from https://perso.uclouvain.be/vincent.blondel/research/louvain.html). To confirm that the modules extracted from the weight matrix corresponded to the assemblies visible in the event raster, we used a standard assembly detection algorithm based on ICA [31].

### Calculation of the eigengap Δλ

The eigengap Δλ was calculated by executing the Louvain method on the weight matrix to estimate the number of assemblies *k* present in the network, and then taking Δλ = Re(λ_*k*_)−Re(λ_*k*+1_), where the λ_*i*_ are the *n* eigenvalues of *w*^*EE*^ such that λ_1_ ≥ ⋯ ≥ λ_*n*_.

### Best match score

We use a modification of the best match algorithm [21, 32] for comparing two sets of assemblies. Given two sets of assemblies $A=\{A_{1},\ldots ,A_{n}\}$ and $B=\{B_{1},\ldots ,B_{m}\}$, we define a similarity measure $\hat{s}\left(c,k\right)$ between assemblies $A_{i}\in A$ and $B_{j}\in B$ as $\hat{s}\left(A_{i},B_{j}\right)=|A_{i}\cap B_{j}\left|/\right|A_{i}\cup B_{j}|$, where |*S*| denotes the cardinality of the set *S*. We then lift the similarity measure $\hat{s}$ between individual assemblies to a similarity measure *s* between full sets of assemblies by setting

$$\begin{array}{c} s\left(A,B\right)=\frac{1}{\left|A|+|B\right|}(\sum_{A\in A}\underset{B\in B}{\text{max}}\hat{s}\left(A,B\right)+\sum_{B\in B}\underset{A\in A}{\text{max}}\hat{s}\left(B,A\right)). \end{array}$$

The measure $\hat{s}$ has the property that if *A* = *B*, then $\hat{s}\left(A,B\right)=1$. Hence if A=B then the normalisation factor ensures s(A,B)=1 as well. Similarly if *A* ∩ *B* = ∅ for all A∈A and B∈B then s(A,B)=0.

### Autosimilarity index

The autosimilarity index for a single simulation was calculated by sampling the set of all existent assemblies $A_{t}$ every 1000 time steps following an initial formation period of 100,000 iterations. For every Δ*t* that was a multiple of 1000, the autosimilarity at Δ*t* was defined as the mean of $s(A_{t_{1}},A_{t_{2}})$ for all pairs *t*_1_ and *t*_2_ such that *t*_2_ − *t*_1_ = Δ*t*.

### Assembly activation statistics in experimental data

A neuron was considered active if its recorded Δ*F*/*F* exceeded its mean by two standard deviations. We generated assembly activity traces as sequences ${\left\{a_{i}^{n}\right\}}_{i}$, where $a_{i}^{n}$ is the number of active neurons in assembly *n* at time-step *i*. Assembly event times were obtained as the local maxima of ${\left\{a_{i}^{n}\right\}}_{i}$ separated by 5 frames where at least 50% of neurons in assembly *n* were active.
